# Detecting Guillain-Barré syndrome caused by Zika virus using systems developed for polio surveillance 

**DOI:** 10.2471/BLT.16.171504

**Published:** 2016-07-05

**Authors:** Nirmal Kandel, Jaya Lamichhane, Rudolf H Tangermann, Guenael RM Rodier

**Affiliations:** a Department of Global Capacities Alert and Response, World Health Organization, avenue Appia 20, 1211 Geneva 27, Switzerland.; b Independent consultant, Geneva, Switzerland.; c Department of Polio Operation and Research, World Health Organization, Geneva, Switzerland.

Zika virus disease is caused by a viral ribonucleic acid (RNA) virus, which is transmitted to humans by mosquitoes of the *Aedes aegypti *species. Around 80% of infections are asymptomatic.^1^ Symptomatic infections are characterized by mild fever lasting from four to seven days, associated with maculopapular rash, arthralgia, conjunctivitis, muscle pain and headache. Until recently, Zika virus disease has never been associated with deaths, intrauterine infections, or congenital anomalies. In 2013 and 2014, during an outbreak in French Polynesia, the disease was linked with Guillain-Barré syndrome.^2^ Zika infection can be established by detection of Zika virus RNA or specific viral antigens in human clinical samples.

It is suspected that over 40 countries had autochthonous Zika virus transmission in 2015 and early 2016.^3^^,^^4^ In some countries, there is a temporal association of Zika virus infections with severe clinical manifestations, particularly Guillain-Barré syndrome and congenital neurological malformations.^3^^,^^4^ In December 2015, officials from the Brazilian Ministry of Health reported 76 patients diagnosed with neurological syndromes, of whom 42 (55%) were confirmed as having Guillain-Barré syndrome.^4^ Similarly, between December 2015 and January 2016, Salvadorian health officials reported 46 patients with Guillain-Barré syndrome, more than 50% of whom had febrile illness lasting between seven to 15 days before onset of the syndrome.^4^ A case-control study conducted in 2013 and 2014 in French Polynesia has shown evidence of Zika virus infections causing Guillain-Barré syndrome.^2^ The French Polynesia study found, in most of the patients, neurological symptoms following Zika virus infections lasted a median of six days.^2^ With increasing evidence of linkages between Guillain-Barré syndrome and Zika virus infection,^2^^–^^4^ it is imperative to enhance Guillain-Barré syndrome surveillance. This can be done using existing surveillance systems like the one for acute flaccid paralysis (AFP) used by polio eradication programmes.^5^

Scientists warn that in view of outbreaks that occurred in Africa, south-east Asia, the Pacific Islands, and the Americas, the disease now has pandemic potential.^6^ In February 2016, the World Health Organization (WHO) declared that the reported clusters of microcephaly and other neurological disorders from the WHO Region of the Americas constituted a Public Health Emergency of International Concern and recommended to enhance surveillance for Zika virus infection.^7^ The *Aedes* species of mosquitoes that transmits the Zika virus and other infections like dengue, chikungunya and yellow fever exists worldwide, posing a high risk for global transmission.^6^ A 2016 modelling study looking at the potential for Zika virus spread predicted substantial international spread by travellers from Brazil to the rest of the world.^8^ Many cases of microcephaly and Guillain-Barré syndrome are now being reported from countries affected by Zika.^2^^–^^4^ Surveillance for timely detection and monitoring of Zika infection and screening for microcephaly and Guillain-Barré syndrome will be essential to guide the public health response.

Governments and other stakeholders use existing AFP surveillance systems in countries to monitor progress towards a global polio eradication goal.^9^ Currently, 91% (177 out of 194) of WHO Member States conduct AFP surveillance. Reporting of AFP in children younger than 15 years is followed by laboratory diagnosis of stool specimens to either confirm polio or identify non-polio AFP cases. Guillain-Barré syndrome cases are classified as non-polio AFP cases. Guillain-Barré syndrome is the most common non-polio cause for AFP. Most countries achieve or surpass the global standard of an annual rate of at least one case of non-polio AFP per 100 000 population of children younger than 15 years. 

In 2015, globally, 99 582 AFP cases among children younger than 15 years, including 72 laboratory-confirmed wild poliovirus cases, were reported. AFP surveillance in countries of the WHO Region of the Americas detected Guillain-Barré syndrome at an annual rate between 0.8 and 1.1 per 100 000 children younger than 15 years.^10^ One study based on a review of published papers between 1980 and 2008 estimated that the global all-age incidence rate for Guillain-Barré syndrome ranges from 1.1 to 1.8 cases per 100 000 people per year. The incidence rate among children younger than 15 years varied from 0.34 to 1.34 cases per 100 000 people per year.^11^ Several reported Zika virus cases from the 2015 and 2016 outbreak have also been linked to Guillain-Barré syndrome.^2^^–^^4^ Further investigation of AFP cases classified as being due to Guillain-Barré syndrome can be a starting point to test for Zika virus.

Existing polio surveillance systems in countries collectively present a platform for global disease detection, monitoring and response.^9^ As these surveillance systems have matured, health officials have increasingly applied them to detect other priority diseases, like measles. A key objective of the *Polio eradication and endgame strategic plan 2013–2018* is to maintain the legacy of the polio eradication programme by ensuring that key assets such as AFP surveillance infrastructure are preserved and used for other epidemic-prone diseases.^5^ The AFP system is fully equipped with trained personnel at national and subnational levels, either with designated surveillance medical officers and/or surveillance focal points, to detect, report and respond to suspected cases of AFP. The system has reporting mechanism from subnational to national level. Trained staff are acquainted – and programmes are equipped – with surveillance protocols for AFP and other diseases. The surveillance system is supported with adequate laboratory facilities from national, regional or international laboratory networks. In many countries governments pay for the system from their national budget, while in others it is supported by international organizations, partners or donors. 

The system provides an infrastructure to monitor the incidence of Guillain-Barré syndrome in children younger than 15 years, and to facilitate surveillance for Zika virus infection in all ages. In addition, symptomatic cases of Zika virus infection present with symptoms like fever and rashes, which are common for measles, dengue and chikungunya; many countries have surveillance systems for such cases, which can be adapted for Zika virus surveillance.^12^ Triangulating the AFP cases with fever and rash cases could increase the sensitivity and specificity of Zika virus infection detection; however, this hypothesis requires further research.

**Fig. 1:**
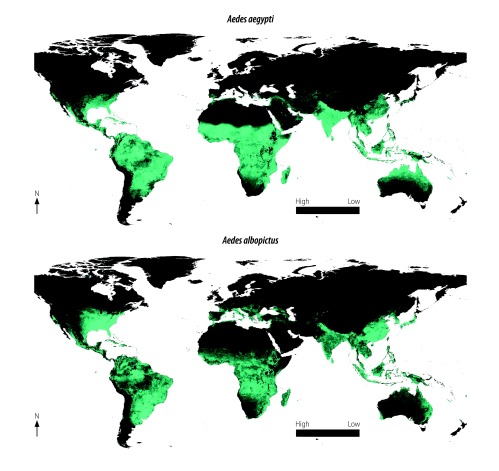
Global distribution of *Aedes aegypti* and *Aedes albopictus *

Source: Adapted with permission.^13^

To enhance monitoring, we suggest that AFP surveillance officers in areas where *Aedes aegypti* is present should immediately notify the detection of clusters of AFP cases diagnosed as Guillain-Barré syndrome, or if there is an increase in the number of AFP/Guillain-Barré syndrome cases above the previous baseline for the area. *Aedes* mosquitoes exist on all continents.^13^ Fig. 1 shows the global distribution of *Aedes aegypti* and *Aedes albopictus*, highlighting the strong likelihood that Zika virus infection will spread across countries and continents.^13^

**Fig. 2:**
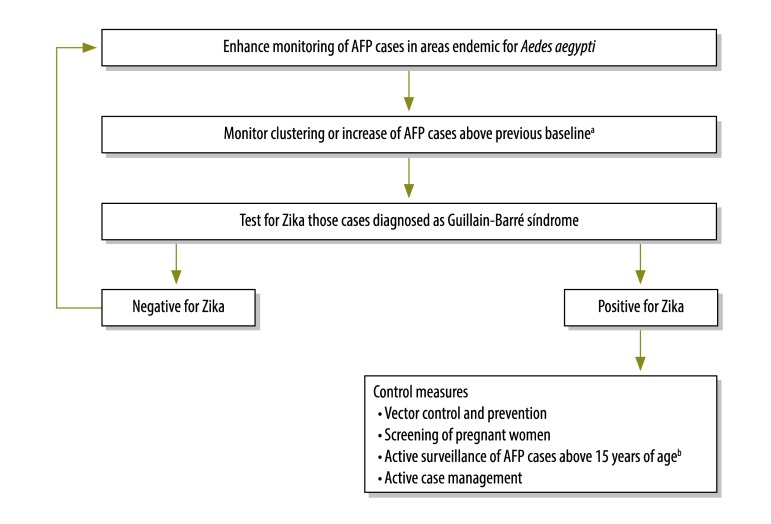
Suggested process for using acute flaccid paralysis surveillance system for Zika virus surveillance

The flow diagram presented in Fig. 2 illustrates the suggested process for using existing AFP surveillance systems for Guillain-Barré syndrome surveillance. We suggest five steps for implementation of the surveillance system in *Aedes* mosquito endemic areas: (i) provide standard case definitions for Zika virus infection to all public health staff working on AFP surveillance, including clinicians working at reporting health facilities in the endemic area for *Aedes* mosquitoes; (ii) raise awareness among all health staff involved in AFP case investigations about the additional measures (testing, referring and reporting) needed for AFP cases provisionally diagnosed as Guillain-Barré syndrome; (iii) investigate all non-polio AFP cases for Guillain-Barré syndrome; (iv) perform Zika virus antibody testing for all confirmed Guillain-Barré syndrome cases or suspected cases reporting symptoms of a possible Zika virus infection, such as fever, rash, arthralgia or conjunctivitis, within three weeks before onset of AFP; and (v) initiate AFP surveillance for all ages, at all tertiary care hospitals or sentinel surveillance hospitals for Guillain-Barré syndrome.

AFP: acute flaccid paralysis.

^a^ Countries have a set yearly AFP target rate to find AFP cases for polio surveillance. This provides the baseline for AFP cases in the population younger than 15 years.

^b^ Although AFP surveillance is for children under 15 years of age, it can be used as a proxy early warning monitoring system for AFP cases in all ages.

Based on the outcome of testing for Zika infection, the following four immediate control measures can be put in place; (i) vector control and prevention; (ii) screening of pregnant women; (iii) active surveillance of any person older than 15 years with AFP; and (iv) active case management. A description of each of the control measures is provided in Box 1.

Box 1 **Control measures following Zika infection testing**

**Vector control and prevention**

This measure refers to any method that aims to limit or eliminate vectors (mosquitoes) which transmit disease pathogens. This type of control measure targets the mosquitoes capable of transmitting Zika virus.

**Screening of pregnant women**

This measure includes a repeated ultrasound of the fetus, preferably between 28 and 30 weeks of pregnancy. In cases where ultrasound results show fetal brain abnormalities and where the woman tests negative for Zika, amniotic fluids are screened for abnormalities, congenital infections and Zika virus.

**Active surveillance of AFP cases older than 15 years**

Targets all cases with acute (rapid) onset of hypotonic (floppy) paralysis in people older than 15 years.

**Active case management**

This measure is a multi-step process of coordination and collaboration on assessment, planning, provision and evaluation of care, as well as advocacy to promote quality care and cost-effective outcomes for Zika patients. The measure also includes provision of supportive services to address the health needs of affected family members.

AFP: acute flaccid paralysis.
